# A model for creating a single stretch injury in murine biarticular muscle

**DOI:** 10.1186/2052-1847-6-14

**Published:** 2014-04-05

**Authors:** Stacey L Brickson, Ronald P McCabe, Adam W Pala, Ray Vanderby

**Affiliations:** 1 Department of Orthopedics and Rehabilitation, University of Wisconsin School of Medicine and Public Health, Madison, WI, USA; 2 Department of Biomedical Engineering, University of Wisconsin School of Medicine and Public Health, Madison, WI, USA

**Keywords:** Eccentric contraction, Experimental model, Muscle injury

## Abstract

**Background:**

We developed a single stretch injury model to create damage near the musculotendinous junction (MTJ) of the gastrocnemius muscle in mice. Our hypothesis was that magnitude of muscle injury could be controlled by stepped shortening of the Achilles tendon (AT) prior to a lengthening contraction. Increased shortening would result in a greater isometric torque deficit and morphological damage 24 hours post-injury.

**Methods:**

Sixteen mice were randomly assigned to sham or injury predicated on stepped increases in AT shortening. The AT was exposed and placed in a customized stainless steel roller-clamp system to achieve a specific level of shortening; 0 mm (resting length), 0.7 mm or 1.4 mm. Plantar flexors were stimulated to tetany with a needle electrode and then actively lengthened at 450°/sec from neutral to 75° of dorsiflexion. Passive and isometric torques were measured pre- and immediately post-injury. Isometric torque was measured again 24 h post-injury. Peak isokinetic torque was recorded during eccentric injury.

**Results:**

Injury resulted in decreased passive and immediate absolute isometric torque only when induced with AT shortening. The percentage of pre-injury isometric torque was significantly lower in the AT shortened groups immediately and 24 h post-injury, but was unaffected by the level of shortening. Relative isometric torque deficits were noted in the 0 mm group only 24 h post-injury. Peak isokinetic torque during injury was similar in all groups. Histological evaluation 24 h post-injury revealed increased morphological damage near the MTJ in the AT shortened groups.

**Conclusion:**

Single stretch with AT shortening created morphological damage near the MTJ and isometric torque deficits immediately and 24 h post-injury, but the magnitude of damage could not be titrated with stepped increases in AT shortening. This model provides an opportunity to utilize transgenic mice in order to elucidate inflammatory mediators that promote regeneration and inhibit fibrosis in order to optimize therapeutic interventions for complete functional recovery.

## Background

Muscle strains are among the most common injuries sustained at work, accounting for 49% of outpatient visits in the military, and comprise 30% of a typical sports medicine practice [1,2]. Incomplete functional recovery and susceptibility to recurrent injury are common sequela of acute muscle strains. Treatment for muscle strain injuries has traditionally included rest, ice, compression and elevation (RICE). Promising new trends to promote complete recovery include novel therapies such as growth factors, gene and stem-cell based therapies [3]. Elucidating effective treatment measures requires a model by which a reproducible, standardized injury can be created that mimics acute muscle strains seen clinically. In this way, various inflammatory mediators that promote regeneration and inhibit fibrosis can be better understood and ultimately optimized for therapeutic intervention.

Acute strains should be distinguished from delayed onset muscle soreness (DOMS) which occurs after exercise involving repeated eccentric contractions and is characterized by microdamage to the muscle*.* Acute muscle strain injuries manifest as severe disruption at the musculotendinous junction (MTJ) primarily in biarticular muscles [1,4]. Fast twitch or Type II fibers appear to be more vulnerable, possibly due to metabolic profile [5,6], higher tensions [7] or relatively shorter optimal lengths of the motor unit [8]. It is well established that muscle strains occur during lengthening of an activated high force generating muscle. Mechanical factors identified as contributing to injury include muscle fiber strain [9-12], peak force [13-15], the product of force and strain [9], contraction velocity [9,14], and initial muscle length [12,16]. However, there is not a consensus as to the relative contribution of these mechanical factors contributing to injury.

Models that have elucidated the mechanical events in eccentric injury have incorporated repetitive lengthening contractions *in situ*[13,15,17] and *in vivo* ranging from 100 to 900 stretches [6,18,19]. For example, 225 stretches to 10% beyond optimal length resulted in 65% force deficit mouse extensor digitorum longus [18] and 900 stretches at 12.5% strain caused a 40% deficit in rabbit TA [11]. It is difficult, however, to induce disruptive muscle damage at or near the MTJ that characterizes acute muscle strains with repetitive eccentric contractions or exercise [20-25]. Furthermore, assessment of damage is confounded with repetitive contractions as force decline is reflective of injury as well as fatigue [11,13].

Single stretch models performed *in situ*[9,26] and *in vitro*[27,28] have also provided powerful insights into the mechanisms for acute strain injuries. Most notably, a minimally invasive standardized single stretch injury method using tendon shortening of the tibialis anterior (TA) muscle in rabbits was developed by Best et al. [29]. Similar *in vivo* single stretch models have been subsequently adapted by other investigators for use in rats [30,31]. Plantar flexion without superimposed tendon shortening produced damage in the TA muscle belly but not at the MTJ [32], which is a hallmark of acute muscle strain injury [1,4,33]. Damage to the gastrocnemius MTJ was observed using ankle excursion in dorsiflexion rather than tendon shortening to manipulate injury [31].

The aim of this study is to evaluate a model for creating a standardized single stretch injury to the *biarticular* gastrocnemius muscle in *mice* using Achilles tendon (AT) shortening to control magnitude of injury. A customized stainless steel roller-clamp system with a bifurcated pin was used to shorten the AT. While the fast twitch fiber composition makes the TA an attractive target muscle, we sought to examine a biarticular muscle as they are more often injured compared to monoarticular muscles [1]. Prior work using tendon shortening [29] provided the unique opportunity to explore the role of inflammatory mediators in exacerbating damage following muscle strain in rabbit TA [34,35]. In the present study, we have sought to adapt this model to the murine species in order to capitalize on knowledge that can be gained through transgenic animals. A single stretch murine injury model would be instrumental in facilitating the exploration and optimization of novel treatments such as stem cell therapies for muscle injury [36].

## Methods

### Instrumentation and experimental apparatus

**Figure 1:**
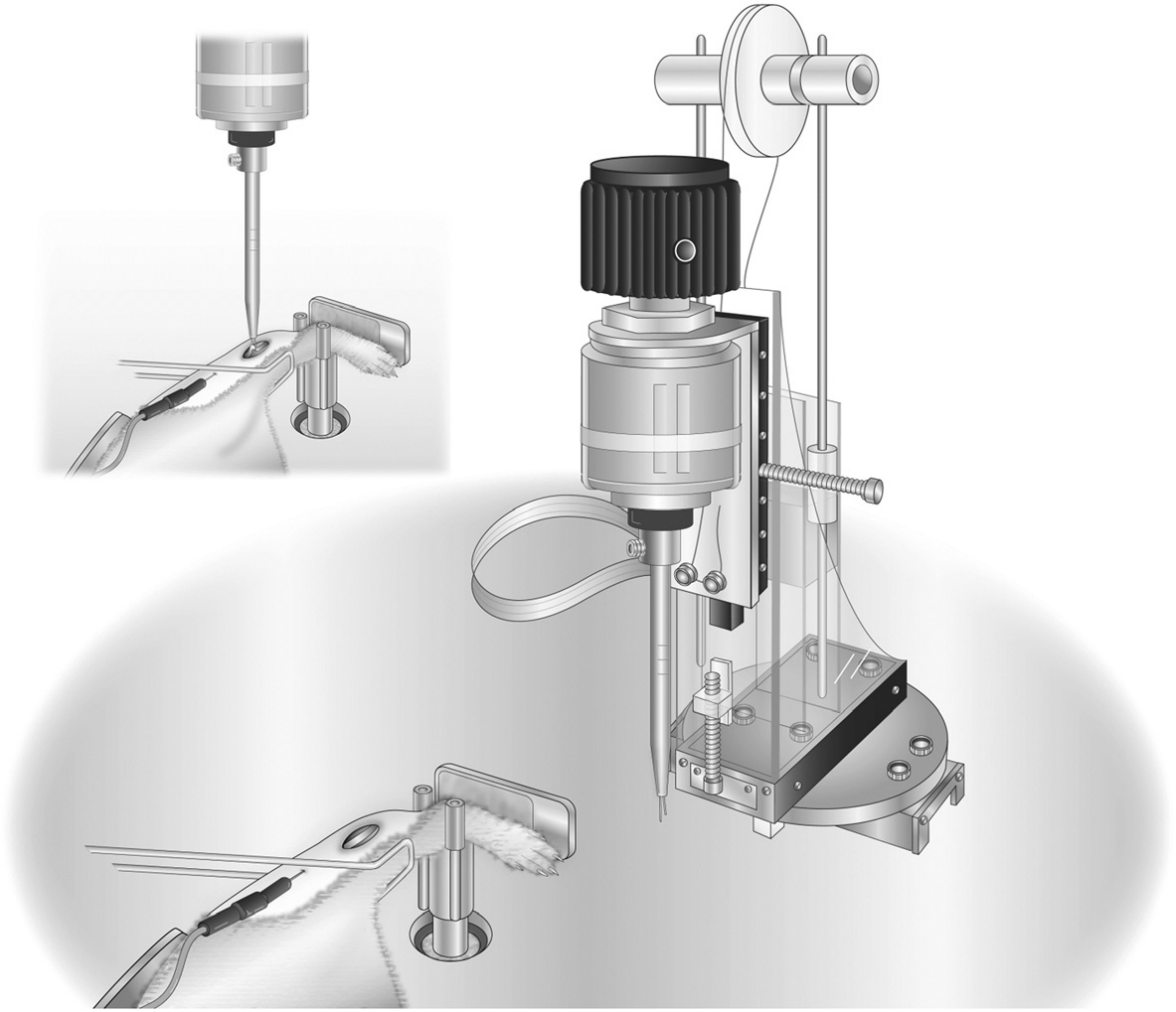
**Experimental apparatus.** An artist’s rendition of the experimental apparatus with the murine specimen’s left hind limb secured in the foot plate and the needle electrode inserted subcutaneously in the popliteal fossa. The inset image additionally depicts the placement of the exposed Achilles tendon in the bifurcated pin.

This system consists of a geared electric motor, torque sensor, angular position sensor, custom designed closed loop control system and nerve stimulator (Figure 1). Torque is measured with a custom fabricated cruxiform torsional load cell with a full scale range of 100 mN · m. Angular position is measured using a custom modified precision potentiometer (Vishay/Spectrol model 138-0-0-103, Vishay Americas, Shelton, CT). The motor is a Faulhaber Model 3863A024C + 38/2S43:1 + X0744 low inertia gear motor (MICROMO, Clearwater, FL). Command signals for the closed loop control system are supplied by a Wavetek arbitrary waveform generator (Model 75, Wavetek Corp, San Diego, CA). Three channels of data (torque, angular displacement and time) are recorded on a Dell computer using a 16 bit Data Translation model DT322 data acquisition board and Data Translation Measure Foundry data acquisition software (Data Translation Inc., Marlboro, MA).

### Experimental injury protocol

This study was performed according to a protocol approved by the University of Wisconsin Institutional Animal Care and Use Committee. Sixteen C57BL/67 male 12 week old mice (26.1 ± 1.7 g, Jackson Laboratory, Bar Harbor, Maine) were assigned to one of four conditions (n = 4 mice, 8 limbs in each group); sham control group or injury group according to one of three incremental levels of AT shortening. The plantar flexor muscles were subjected to a single lengthening contraction without shortening (0 mm), AT shortening of 0.7 mm or 1.4 mm, corresponding to approximately 10% and 20% of resting AT length, respectively [37], consistent with the percentage of TA shortening in the rabbit model [29,38]. Tendon shortening was achieved using a bifurcated pin system connected to a precision potentiometer. To calibrate the shortening, a fine thread was inserted between the two pins and connected to a precision rectilinear potentiometer. The bifurcated pin potentiometer shaft was then rotated, while its output voltage and the rectilinear potentiometer output voltage were acquired with a data acquisition system. Collected data were converted into a displacement (shortening) versus voltage (theta) curve. A sixth order polynomial was then fit to the curve over the range of 0 to 4 mm with a resultant R^2^ of 0.9999.

Mice were anesthetized with isoflurane and a 2-mm incision made to expose the AT. A custom splint was placed over the femur and tibia to ensure the knee joint angle remained at 0° throughout the test. The animal was placed side lying in a metal half cylinder warming unit to maintain body temperature at 37°C. Care was taken to ensure that the animal was positioned so that the axis of ankle joint rotation was aligned with the axis of rotation of the loading frame. The foot was secured to a foot plate and locked in a neutral position (tibiotarsal angle of 90°) perpendicular to the tibia. Side lying was selected to eliminate the effect of gravity on joint torque. The ankle was aligned between two pins immediately anterior and posterior to the axis of rotation at the ankle joint. Brass cylinders of various sizes were machined to fit over the pin to allow the ankle to remain secured between the pins without impeding motion throughout the physiological range.

#### Pre-injury passive peak and isometric torque measurements

With the animal secured in the apparatus, the ankle was rotated from neutral through a 75° arc into dorsiflexion at an angular velocity was 450°/sec and the peak passive torque recorded (Additional file 1: Figure S1). The load frame was then fixed so that isometric torque could be recorded. The plantar flexors were stimulated to tetany (2.8 mA pulse train, 100Hz, 2.0 ms pulse width and triangular wave) with a needle electrode inserted subcutaneously in the popliteal fossa. To determine the simulating frequency, plantar flexor muscles were stimulated at 50, 75, 100 and 150Hz. Isometric torque did not increase from 100 to 150Hz and therefore 100Hz was selected. Two isometric torques were recorded with 3-min rest intervals between tests and the average torque was calculated.

#### Isokinetic torque measurements

**Figure 2:**
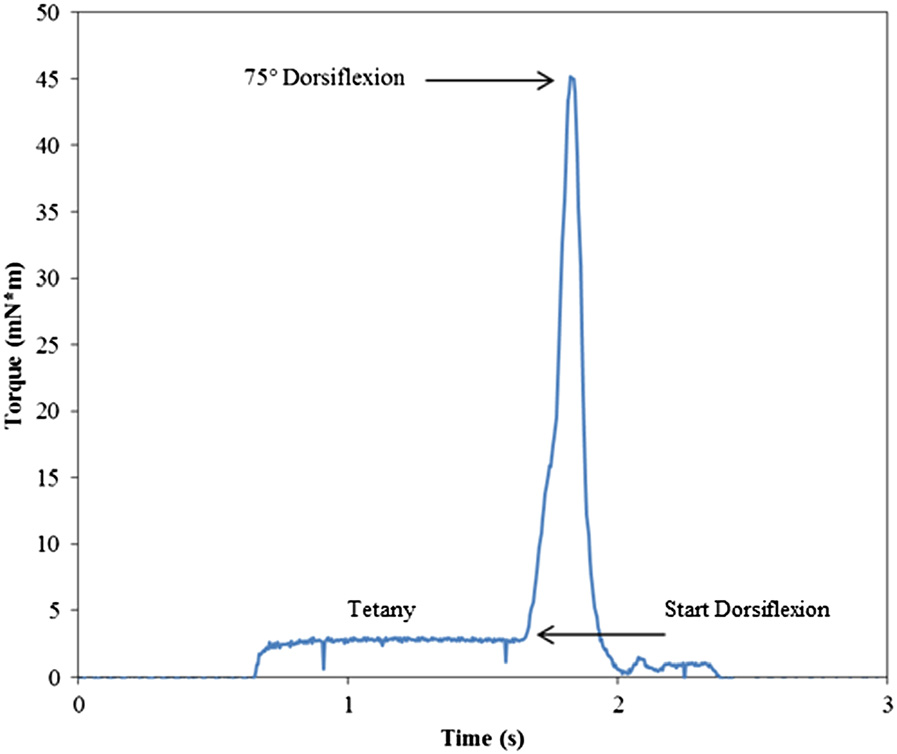
**Torque-time plot.** A representative injury waveform from the 1.4 mm AT shortening group is shown wherein tetany is achieved and subsequent rotation of the foot plate (75° dorsiflexion) occurs upon a 1.0 s delay of electrical stimulation. Isometric torque, peak isokinetic torque and waveform were similar for all groups. Peak torque is reached at the end range of motion.

The AT was visualized and placed in a customized stainless steel roller-clamp system and left at resting length (0 mm, no shortening), or shortened 0.7 mm or 1.4 mm. The bifurcated pin was tapered to allow ease of accepting the AT into the roller-clamp initially while prohibiting slippage once the tendon is turned to wrap back onto itself, shortening it 0.7 mm or 1.4 mm from resting length. A 1.0-s trigger delay between the initial muscle stimulation and ankle rotation ensured the achievement of complete muscle tetany. Muscle stimulation ceased immediately upon the footplate returning to neutral. In all groups, the angular velocity was 450°/sec and the ankle was rotated from neutral through a 75° arc into dorsiflexion and returned to neutral, at which time stimulation ceased. Torque-time plots were analyzed for ankle joint torque produced during muscle tetany and the peak joint torque at the end range of dorsiflexion (Figure 2). A sham group was included whereby the AT was exposed and ankle rotation occurred without stimulation or AT shortening (passive lengthening).

#### Post-injury peak passive and isometric torque measurements

The tendon was released from the roller-clamp system following injury isometric torque was immediately assessed as described above. The needle electrode was removed, the footplate unlocked, and peak passive torque was recorded. Incisions were closed with Vetbond and animals were returned to their cages for 24 h. Isometric torque measurements were again recorded 24 h post-injury in the absence of AT shortening.

### Histology

The AT was transected and the triceps surae muscle resected. The gastrocnemius was isolated, dissected and frozen in isopentane cooled in liquid nitrogen and stored at -80°C. Serial 10 μm cross-sections immediately adjacent to the tendon were obtained using a cryostat (Leica/Jung CM 1800 model, IMEB, Inc, San Marcos, CA) and subsequently stained with hematoxylin and eosin (H&E). Injury was identified as the region with greatest disruption of fibers and nuclei accumulation. Damage was characterized in a semi-quantitative manner by counting multiple nuclei accumulation (expressed as nuclei/mm^2^) 24 h post-injury. The cellular response to injury infers an increase in nuclei above baseline, but is not specific for myonuclei, neutrophils, macrophages and fibroblasts. In order to establish the location and extent of injury, damage was investigated in a spatially sensitive manner in the 1.4 mm shortening group. Tissue was carefully cut into 1 mm segments (0-1 mm, 1-2 mm, 2-3 mm and 3-4 mm) starting at the MTJ and continuing proximally through the muscle belly. As damage was localized immediately adjacent to the MTJ, only this area was viewed for subsequent groups. Micrographs were collected using a camera-assisted microscope (Nikon Eclipse microscope model E6000, Nikon Instruments, Inc., Mellville, NY with an Olympus camera, model DP70, Olympus Imaging America, Inc., Center Valley, PA). Five sections from each muscle were viewed and two computer images (0.25 mm × 0.25 mm) were captured per section and viewed using Image J.

### Statistics

Means and standard errors were calculated. The paired Students *t* test was used to compare the passive torques before and immediately after injury. Dunnett’s multiple comparison test was performed when absolute isometric torques immediately and 24 h post-injury were compared with pre-injury torques. Two-way analysis of variance (ANOVA) and the Bonferroni post hoc test were used to compare the percentage of pre-injury isometric torque between the groups at both time points. Statistical analyses were carried out using KaleidaGraph, version 4.03 (Synergy Software, Inc., Reading, PA).

## Results

### Passive torque

**Figure 3:**
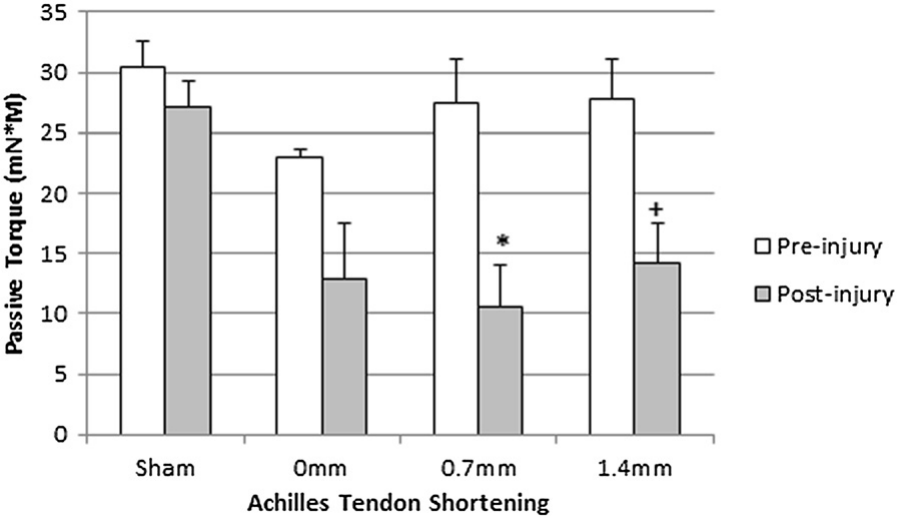
**Passive torques pre- and post-injury.** Passive torques before and after injury with different levels of AT shortening. Passive torque before injury (open bar) was compared in a pairwise fashion with that immediately after injury (shaded bar). Values were expressed as mean ± SEM. +P < 0.05, *P < 0.01 versus pre-injury.

The peak passive torque during 75° of dorsiflexion was measured in the absence of tendon shortening in all groups prior to and immediately following injury. Passive torque was compared in a pairwise fashion with the pre-injury value. Significant decreases were observed in the 0.7 mm (P < 0.01) and 1.4 mm (P < 0.05) AT shortened groups (Figure 3).

### Isometric and peak isokinetic torques

**Figure 4:**
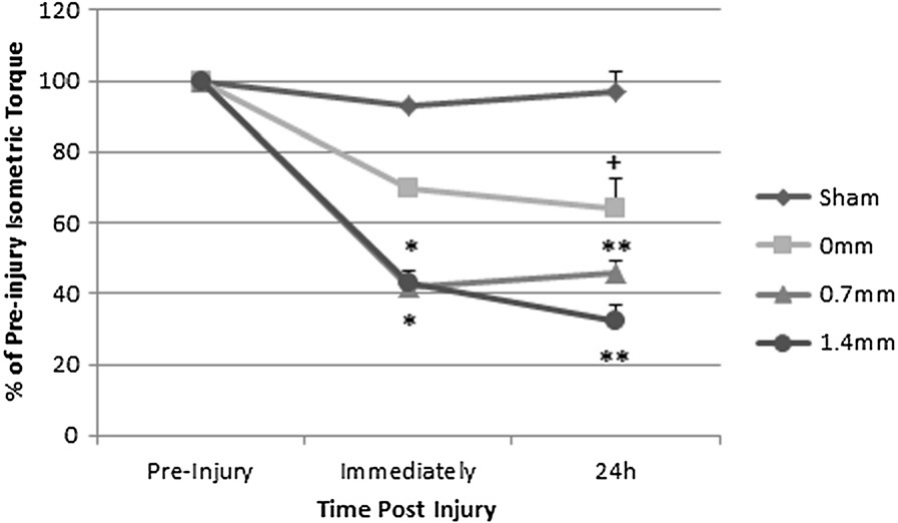
**Isometric torques expressed as a percentage of pre-Injury torque.** Relative difference in torques (expressed as percentage of pre-injury) between the 4 groups measured immediately and 24 h post-injury and analyzed with a two-way ANOVA. Values were expressed as mean ± SEM. Both AT shortened groups displayed deficits immediately (**P < 0.001) and 24 h post-injury (*P < 0.01) compared to all time points for the sham group. Deficits in the 0 mm group were evident only 24 h post-injury (+P < 0.05) compared to the sham group.

Isometric torque measures were recorded pre-injury and repeated immediately and 24 h post-injury. As shown in Table 1, multiple-comparisons of absolute isometric torque were significantly lower immediately and 24 h post-injury in the 0.7 mm (P < 0.01) and 1.4 mm (P < 0.001) AT shortening groups. The 0 mm group showed a trend for a torque deficit at 24 h (P = 0.07). The relative difference in isometric torque (percentage of pre-injury torque) between groups was examined immediately and 24 h post-injury and found to be significant (Figure 4). A substantial decrease in the percentage of isometric torque was observed in both the 0.7 mm and 1.4 mm AT shortened groups immediately (P < 0.01) and 24 h post-injury (P < 0.001) compared to all sham time points. The 0 mm injury group showed a trend for relative isometric torque deficit immediately (P = 0.07), and was significant at 24 h from all sham time points (P < 0.05). Peak isokinetic torque occurred at the end range of dorsiflexion and reflects the passive and active torques. Peak torque was unaffected by AT shortening and reached 40.06 ± 11.21, 39.49 ± 9.89 and 39.32 ± 9.67 mN · m in the three injured groups, respectively (mean ± SD). The isometric to peak isokinetic torque ratio is consistent with those reported by Best et al. in rabbit TA [29] using a similar model.

**Table 1:** Time course for isometric torque following single stretch injury

	**Pre-injury**	**Immediately post-injury**	**24h Post-injury**
Sham	7.22 ± 0.05	7.7 ± 0.72	7.52 ± 0.55
0mm	6.95 ± 2.18	4.81 ± 1.34	3.94 ± 2.08
0.7mm	6.95 ± 1.92	2.49 ± 0.93^a^	2.21 ± 0.46^a^
1.4mm	7.51 ± 0.79	3.12 ± 1.01^b^	1.65 ± 0.54^b^

Values are presented in mN · m as mean ± SD. Isometric torque was measured pre-injury, immediately and 24 h post-injury and analyzed using Dunnett’s multiple comparison test. ^a^P < 0.01, ^b^P < 0.001 versus pre-injury torque.

### Histology

**Figure 5:**
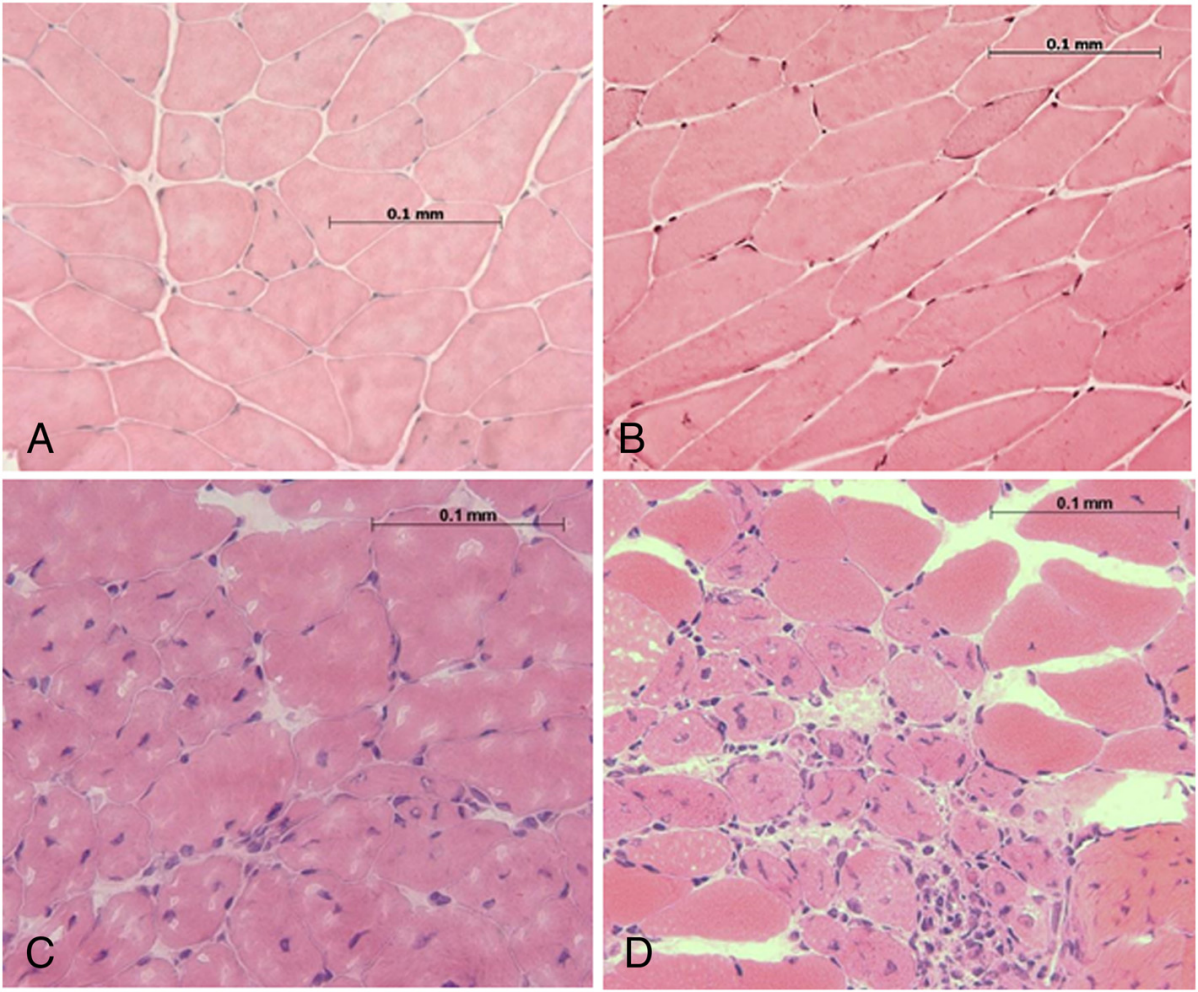
**Histology.** Representative H&E stained cross-sections are shown for the **(A)** uninjured control, **(B)** 0 mm, **(C)** 0.7 mm and **(D)** 1.4 mm groups. AT shortened groups are characterized by more nuclei and fiber disruption.

**Figure 6:**
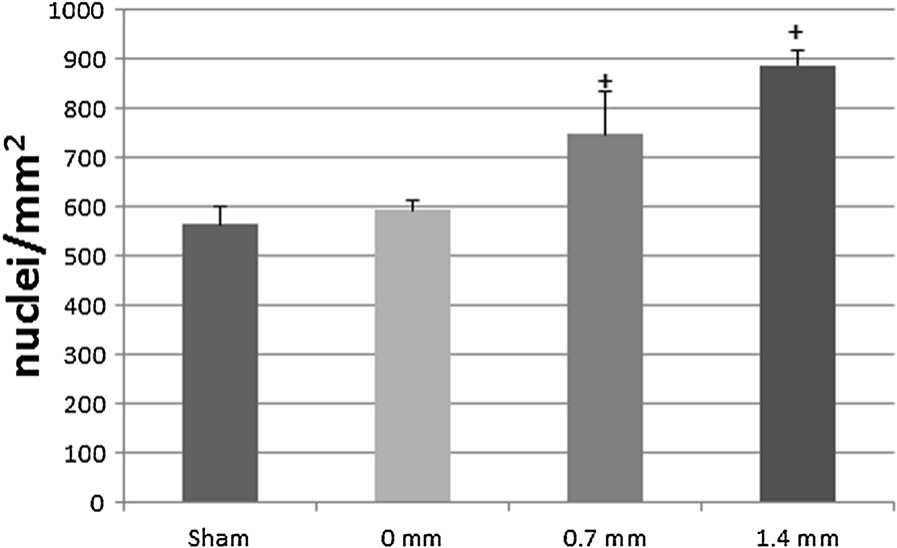
**Morphological damage.** Nuclei counts (nuclei/mm^2^) obtained using H&E stained cross-sections are shown for the gastrocnemius muscle immediately adjacent to the MTJ. Damage 24 h post-injury was appreciated in the two groups with AT shortening (+P < 0.05), but the stepped increase in AT shortening from 0.7 mm to 1.4 mm did not result in greater damage.

Damage was characterized by disruption of fibers and described in a semi-quantitative manner by counting multiple nuclei accumulation 24 h post-injury. Damage between groups was assessed immediately adjacent to the MTJ and found to be significant in injured groups with AT shortening (Figure 5). Nuclei accumulation in the injured gastrocnemius without AT shortening (0 mm) was 565 ± 38 nuclei/mm^2^ and not different from uninjured controls (594 ± 20 nuclei/mm^2^). AT shortening to 0.7 or 1.4 mm resulted in increased cellularity above uninjured and 0 mm groups to 748 ± 86 and 888 ± 28 nuclei/mm^2^, respectively, but there was not a further increase with the stepped increase in AT shortening (P < 0.05) (Figure 6).

## Discussion

We have developed a single stretch injury model for murine gastrocnemius via shortening the AT that results in damage near the MTJ, similar to the location of damage in acute muscle strains noted clinically. Injury was defined by morphological damage and isometric torque deficits immediately and 24 h post-injury. A murine model for single stretch may provide a useful tool for utilizing transgenic animals to elucidate the role of various inflammatory mediators in order to facilitate regeneration and minimize fibrosis.

From a clinical perspective, the severity of muscle damage is perhaps best characterized by deficits in force production or torque [13,14,39-41]. Torque is the product of the force produced by a muscle or muscle group and the moment arm of the muscle(s) attachment. Absolute isometric torque measurements recorded using a torsional load cell dropped in AT shortened injured groups immediately post-injury and were consistent with values from other reports [42,43], but were not significant in the 0 mm injured group. AT shortening was prerequisite to elicit significant deficits in absolute isometric torque immediately and 24 h post-injury using multiple comparison analysis. Deficits expressed as the percentage of pre-injury isometric torque between groups revealed that AT shortening was prerequisite to elicit significant deficits immediately post-injury. Without AT shortening, deficits in percentage of isometric torque were only significant 24 h following injury. We propose that the mechanical damage elicited without AT shortening was sufficient to prompt further damage through the inflammatory cascade, resulting in deficits that persisted 24 h post-injury. AT shortening resulted in increased mechanical damage which manifested as immediate isometric deficits and those deficits were magnified 24 h post-injury. Cumulative effects of the initial stretch injury with resultant cascade of chemical and metabolic factors have been proposed to exacerbate mechanical deficits 24 h post-injury [11,13,18,34,35,40]. This study selected 24 h for sacrifice in order to capture the peak time point for mechanical damage; however, a time course to elucidate the duration of mechanical deficits would be an interesting contribution.

Persistent force deficits have been documented up to three days following a single stretch injury without superimposed AT shortening by modulating range of motion from 10° to 25° of dorsiflexion in rat gastrocnemius muscle [31]. One possibility for this disparity in damage between species and models may be the degree of fiber strain during stretch. In a recent elegant *in vivo* study by Butterfield and Herzog [12], excursion of the muscle tendon unit (MTU) was held constant while the timing of activation and starting length were altered in rabbit TA muscle for 50 lengthening contractions. Maximal fiber strain was measured directly and was found to increase with preactivation and starting muscle length. The magnitude of fiber strain was shown to be more important than muscle force or MTU dynamics in contributing to injury. While the precise relationship between fiber strain and MTU strain has not been established, increased compliance of the contractile element has been shown [44] discrediting the direct one to one relationship assumed in *in vitro* and *in situ* injury models. MTU lengthening occurs paradoxically with fiber shortening during ambulation in cat soleus muscle, indicating that MTU strains exceed muscle fiber strains [45]. By shortening the AT prior to lengthening contraction, the ability of the MTU to minimize fiber strain might have been attenuated, thus creating muscle injury.

The viscoelastic nature of muscle must be considered in minimizing the variability of mechanical testing [46]. Passive properties of muscle have been shown to contribute nearly half of total force of plantar flexion in mice [9]. Our data suggests that the passive component contributed to more than half of peak torque during active lengthening. In permeabilized rat soleus fibers, passive tension reached 80% of maximum isometric force during a first passive stretch, and this decreased to approximately 50% with preconditioning [47]. Our data agrees with passive tension contributing to the majority of peak isokinetic torque, but this did not decline following one passive stretch to precondition the muscle.

Passive torque was substantially decreased immediately following injury in groups with AT shortening but was not affected by the level of shortening. Elastic myofilaments contribute to passive stability, with titin serving as a spring activated by calcium binding to increase stiffness during contraction [48]. These findings suggests that passive torque generated by parallel elastic structures such as titin or the extracellular matrix were not incrementally damaged in accordance with AT shortening [49].

Histological verification of damage has been touted as the “gold standard” as it allows visualization of morphological changes that can be appreciated qualitatively [50]. Quantification of histological damage is more difficult, but has been shown to be reliable when there is an established set of criteria to quantify muscle damage [51], such as inflammatory cell infiltration on H&E sections [15]. Disruption of fibers and accumulation of nuclei within the interstitial space or myofibers was not appreciably increased in the 0 mm group over the sham control. AT shortening was prerequisite to elicit damage visualized by morphological changes 24 h following injury, illustrating a similar trend with mechanical deficits. It should be noted that although morphological changes have been correlated with loss of contractile force [19], a causal relationship has not been established for either magnitude or time course [39]. Although persistent isometric torque deficit and morphological damage were only evident upon AT shortening, we were unable to precisely titrate the magnitude of damage with stepped increases in AT shortening.

Developing a murine *in vivo* model for acute muscle strain that results in damage near the MTJ seen clinically has proven challenging, possibly due to active and passive sarcomere length heterogeneity [47,52]. To our knowledge, this is the first minimally invasive *murine* model able to create damage near the MTJ with a single stretch of a biarticular muscle while retaining the intact MTU. Pioneering research incorporating single stretch injury models *in situ* and *in vitro* have elucidated mechanical factors contributing to damage [9,26-28]. Less invasive injury models that did not require tendon detachment were created for rabbit TA [29] and subsequently adapted for rat TA [30] and gastrocnemius [31]. This model provides an opportunity to utilize genetically engineered mice to expand the exploration of factors involved in tissue healing.

There are several limitations inherent to our study. While shortening of the AT is minimally invasive and an improvement from *in situ* models whereby the MTJ is detached, it is still a non-physiologic model. Furthermore, we were unable to titrate the level of damage by stepped increases in AT shortening. The contribution of this study is to provide a model for single stretch injury, not to elucidate the mechanical events creating damage. We speculated that shortening of the AT dampened the ability of the MTU to minimize fiber strain, but strain was not measured. In future work, the method of electrical stimulation should be optimized. Isometric tetanic force has be shown to be two times lower with tibial nerve stimulation compared to skin stimulation [31]. A needle electrode does not permit isolation of the target gastrocnemius muscle, but results in the contraction of the triceps surae and deep flexors. Subsequently, isometric torque measurements reflect the contribution of the entire posterior compartment musculature. Finally, while morphological damage confirms injury [13,15], quantitative measures are difficult. Cellular response to injury was non-specific as we only evaluated nuclei using H&E stain. Specific cell type identification using immunohistochemistry or assays to quantify inflammatory genes or proteins would have provided more sensitive information about the inflammatory response.

## Conclusions

We have created a minimally invasive murine model for single stretch injury that results in damage localized near the MTJ of a biarticular muscle. Shortening of the Achilles tendon was prerequisite for incurring damage as identified by immediate absolute and relative isometric torque deficits. Morphological damage was noted 24 h post-injury in the AT shortened groups, along with a further decline in relative isometric torque. This model will provide an opportunity to utilize transgenic mice to further elucidate the role of various inflammatory mediators involved in fibrosis and regeneration.

## Competing interests

The authors declare that they have no competing interests.

## Authors’ contributions

SB designed the study, performed the injury testing, mechanical data collection, data analysis and manuscript preparation. RM designed the isokinetic device and software. AP performed the histology and helped with software and optimization of the testing. RV provided use of his laboratory. All the authors read and approved the final manuscript.

## Pre-publication history

The pre-publication history for this paper can be accessed here:

http://www.biomedcentral.com/2052-1847/6/14/prepub

## Supplementary Material

Additional file 1: Figure S1 An angle versus time plot illustrating the footplate traveling through 75° of dorsiflexion. Click here for file

## References

[B1] Garrett WE Jr Muscle strain injuries Am J Sports Med 1996 24 S2 S8 10.1177/036354659602400102 8947416

[B2] Jones BH Canham-Chervak M Canada S Mitchener TA Moore S Medical surveillance of injuries in the u.s. Military descriptive epidemiology and recommendations for improvement Am J Prev Med 2010 38 S42 S60 10.1016/j.amepre.2009.10.014 20117600

[B3] Huard J Li Y Fu FH Muscle injuries and repair: current trends in research J Bone Joint Surg 2002 84 822 832 12004029

[B4] De Smet AA Best TM MR imaging of the distribution and location of acute hamstring injuries in athletes Am J Roentgenol 2000 174 393 399 10.2214/ajr.174.2.1740393 10658712

[B5] Friden J Lieber RL Segmental muscle fiber lesions after repetitive eccentric contractions Cell Tissue Res 1998 293 165 171 10.1007/s004410051108 9634608

[B6] Lieber RL Friden J Selective damage of fast glycolytic muscle fibers with eccentric contraction of the rabbit tibialis anterior Acta Physiol Scand 1988 133 587 588 10.1111/j.1748-1716.1988.tb08446.x 3227940

[B7] Appell HJ Soares JM Duarte JA Exercise, muscle damage and fatigue Sports Med 1992 13 108 115 10.2165/00007256-199213020-00006 1561506

[B8] Brockett CL Morgan DL Gregory JE Proske U Damage to different motor units from active lengthening of the medial gastrocnemius muscle of the cat J Appl Physiol 2002 92 1104 1110 11842046 10.1152/japplphysiol.00479.2001

[B9] Brooks SV Faulkner JA Severity of contraction-induced injury is affected by velocity of stretch only during stretches of large strain J Appl Physiol 2001 91 661 666 11457778 10.1152/jappl.2001.91.2.661

[B10] Lieber RL Friden J Mechanisms of muscle injury gleaned from animal models Am J Phys Med Rehabil 2002 81 S70 S79 10.1097/00002060-200211001-00008 12409812

[B11] Lieber RL Friden J Muscle damage is not a function of muscle force, but active muscle strain J Appl Physiol 1993 74 510 526 8458765 10.1152/jappl.1993.74.2.520

[B12] Butterfield TA Herzog W Effect of altering starting length and activation timing of muscle on fiber strain and muscle damage J Appl Physiol 2006 100 1489 1498 10.1152/japplphysiol.00524.2005 16397062

[B13] McCully KK Faulkner JA Injury to skeletal muscle fibers of mice following lengthening contractions J Appl Physiol 1985 59 119 126 4030553 10.1152/jappl.1985.59.1.119

[B14] Warren GL Hayes DA Lowe DA Armstrong RB Mechanical factors in the initiation of eccentric contraction-induced injury in rat soleus muscle J Physiol 1993 464 457 475 8229813 10.1113/jphysiol.1993.sp019645 PMC1175396

[B15] McCully KK Faulkner JA Characteristics of lengthening contractions associated with injury to skeletal muscle fibers J Appl Physiol 1986 61 293 299 3733615 10.1152/jappl.1986.61.1.293

[B16] Talbot JA Morgan DL The effects of stretch parameters on eccentric exercise-induced damage to toad skeletal muscle J Muscle Res Cell Motil 1998 19 237 245 10.1023/A:1005325032106 9583364

[B17] Lieber RL Woodburn TM Friden J Muscle damage induced by eccentric contractions of 25% strain J Appl Physiol 1991 70 2498 2507 1885443 10.1152/jappl.1991.70.6.2498

[B18] Brooks SV Faulkner JA Contraction-induced injury: recovery of skeletal muscles in young and old mice Am J Physiol 1990 258 C436 C442 2316632 10.1152/ajpcell.1990.258.3.C436

[B19] Lieber RL Thronell L-E Friden J Muscle cytoskeletal disruption occurs within the first 15 min of cyclic eccentric contraction J Appl Physiol 1996 80 278 284 10.1063/1.362816 8847315

[B20] Armstrong RB Ogilvie RW Schwane JA Eccentric exercise-induced injury to rat skeletal muscle J Appl Physiol 1983 54 80 93 6826426 10.1152/jappl.1983.54.1.80

[B21] Cutlip RG Geronilla KB Baker BA Kashon ML Miller GR Schopper AW Impact of muscle length during stretch-shortening contractions on real-time and temporal muscle performance measures in rats in vivo J Appl Physiol 2004 96 507 516 10.1152/japplphysiol.00046.2003 14555680

[B22] Friden J Changes in human skeletal muscle induced by long term eccentric exercise Cell Tissue Res 1984 236 365 372 6733763 10.1007/BF00214240

[B23] Geronilla KB Miller GR Mowrey KF Wu JZ Kashon ML Brumbaugh K Reynolds J Hubbs A Cutlip RG Dynamic force responses of skeletal muscle during stretch-shortening cycles Eur J Appl Physiol 2003 90 144 153 10.1007/s00421-003-0849-8 14504946

[B24] Ingalls CP Wenke JC Nofal T Armstrong RB Adaptation to lengthening contraction-induced injury in mouse muscle J Appl Physiol 2004 97 1067 1076 10.1152/japplphysiol.01058.2003 15121748

[B25] McHugh MP Pasiakos S The role of exercising muscle length in the protective adaptation to a single bout of eccentric exercise Eur J Appl Physiol 2004 93 286 293 10.1007/s00421-004-1196-0 15338218

[B26] Brooks SV Zerba E Faulkner J Injury to muscle fibers after single stretches of passive and maximally stimulated muscle in mice J Appl Physiol 1995 488 459 469 10.1113/jphysiol.1995.sp020980 PMC1156684 8568684

[B27] Lynch GS Faulkner JA Contraction-induced injury to single muscle fibers: velocity of stretch does not influence the force deficit Am J Physiol 1998 275 C1548 C1554 9843716 10.1152/ajpcell.1998.275.6.C1548

[B28] Macpherson PC Schork MA Faulkner JA Contraction-induced injury to single fiber segments from fast and slow muscles of rats by single stretches Am J Physiol 1996 271 C1438 C1446 8944625 10.1152/ajpcell.1996.271.5.C1438

[B29] Best TM McCabe RP Corr DT Vanderby R Evaluation of a new method to create a standardized muscle stretch injury Med Sci Sports Exerc 1998 30 200 205 9502346 10.1097/00005768-199802000-00005

[B30] Lovering RM Hakim M Moorman CT 3rd De Deyne PG The contribution of contractile pre-activation to loss of function after a single lengthening contraction J Biomech 2005 38 1501 1507 10.1016/j.jbiomech.2004.07.008 15922761 PMC4489540

[B31] Song H Nakazato K Nakajima H Effect of increased excursion of the ankle on the severity of acute eccentric contraction-induced strain injury in the gastrocnemius: an in vivo rat study Am J Sports Med 2004 32 1263 1269 10.1177/0363546503262199 15262652

[B32] Lovering RM McMillan AB Gullapalli RP Location of myofiber damage in skeletal muscle after lengthening contractions Muscle Nerve 2009 40 589 594 10.1002/mus.21389 19760787 PMC3521509

[B33] Jarvinen TA Jarvinen TL Kaariainen M Kalimo H Jarvinen M Muscle injuries: biology and treatment Am J Sports Med 2005 33 745 764 10.1177/0363546505274714 15851777

[B34] Brickson S Hollander J Corr DT Ji LL Best TM Oxidant production and immune response following stretch injury in skeletal muscle Med Sci Sports Exerc 2001 33 2010 2015 11740292 10.1097/00005768-200112000-00006

[B35] Brickson S Ji LL Schell K Olabisi R Schneider BSP Best TM M1/70 attenuates blood-borne neutrophil oxidants, activation, and myofiber damage following stretch injury J Appl Physiol 2003 95 969 976 12730143 10.1152/japplphysiol.00005.2003

[B36] Quintero AJ Wright VJ Fu FH Huard J Stem cells for the treatment of skeletal muscle injury Clin Sports Med 2009 28 1 11 10.1016/j.csm.2008.08.009 19064161 PMC2630112

[B37] Zhou Z Yang ZM Xie HQ Preliminary study of cryopreservation of tissue engineered tendon Zhongguo Xiu Fu Chong Jian Wai Ke Za Zhi 2002 16 295 299 12569797

[B38] Alder AB Crawford GNC Edwards RG The growth of the muscle tibialis anterior in the normal rabbit in relation to the tension-length ratio Proc R Soc Lond B Biol Sci 1958 148 207 216 10.1098/rspb.1958.0014 13518135

[B39] Newham DJ McPhail G Mills KR Edwards RHT Ultrastructural changes after concentric and eccentric contractions of human muscle J Neurol Sci 1983 61 109 122 10.1016/0022-510X(83)90058-8 6631446

[B40] Zerba E Komorowski TE Faulkner J Free radical injury to skeletal muscles of young, adult, and old mice Am J Physiol 1990 258 C429 C435 2316631 10.1152/ajpcell.1990.258.3.C429

[B41] Warren GL Ingalls CP Lowe DA Armstrong RB What mechanisms contribute to the strength loss that occurs during and in the recovery from skeletal muscle injury? J Orthop Sports Phys Ther 2002 32 58 64 10.2519/jospt.2002.32.2.58 11838581

[B42] Hesselink RP Gorselink M Schaart G Wagenmakers AJ Kamphoven J Reuser AJ Van Der Vusse GJ Drost MR Impaired performance of skeletal muscle in alpha-glucosidase knockout mice Muscle Nerve 2002 25 873 883 10.1002/mus.10125 12115977

[B43] Warren GL Ingalls CP Armstrong RB A stimulating nerve cuff for chronic in vivo measurements of torque produced about the ankle in the mouse J Appl Physiol 1998 84 2171 2176 9609814 10.1152/jappl.1998.84.6.2171

[B44] Butterfield TA Herzog W Quantification of muscle fiber strain during in vivo repetitive stretch-shortening cycles J Appl Physiol 2005 99 593 602 10.1152/japplphysiol.01128.2004 15790684

[B45] Griffiths RI Shortening of muscle fibres during stretch of the active cat medial gastrocnemius muscle: the role of tendon compliance J Physiol 1991 436 219 236 2061831 10.1113/jphysiol.1991.sp018547 PMC1181502

[B46] Best TM McElhaney J Garrett WE Jr Myers BS Characterization of the passive responses of live skeletal muscle using the quasi-linear theory of viscoelasticity J Biomech 1994 27 413 419 10.1016/0021-9290(94)90017-5 8188722

[B47] Palmer ML Claflin DR Faulkner JA Panchangam A Non-uniform distribution of strain during stretch of relaxed skeletal muscle fibers from rat soleus muscle J Muscle Res Cell Motil 2011 32 39 48 10.1007/s10974-011-9250-0 21710358 PMC3184522

[B48] Bagni MA Cecchi G Colombini B Colomo F A non-cross-bridge stiffness in activated frog muscle fibers Biophys J 2002 82 3118 3127 10.1016/S0006-3495(02)75653-1 12023235 PMC1302100

[B49] Horowits R Passive force generation and titin isoforms in mammalian skeletal muscle Biophys J 1992 61 392 398 10.1016/S0006-3495(92)81845-3 1547327 PMC1260255

[B50] Bar PR Reijneveld JC Wokke JHJ Jacobs SCJM Bootsma AL Salmons S Muscle damage induced by exercise: nature, prevention and repair Muscle Damage 1997 Oxford UK: Oxford University Press 1 27

[B51] Prisby RD Reyes RA Eason JM Nelson AG Criteria for assessing skeletal muscle damage from electron micrographs Med Sci Sports Exerc Suppl 2001 33 S123

[B52] Patel TJ Das R Friden J Lutz GJ Lieber RL Sarcomere strain and heterogeneity correlate with injury to frog skeletal muscle fiber bundles J Appl Physiol 2004 97 1803 1813 10.1152/japplphysiol.00505.2003 15208284

