# Supplementary figures and images for: A model for creating a single stretch injury in murine biarticular muscle
Source: BMC Sports Sci Med Rehabil. 2014 Apr 5;6:14. doi: 10.1186/2052-1847-6-14 (PMC4022121; doi:10.1186/2052-1847-6-14)

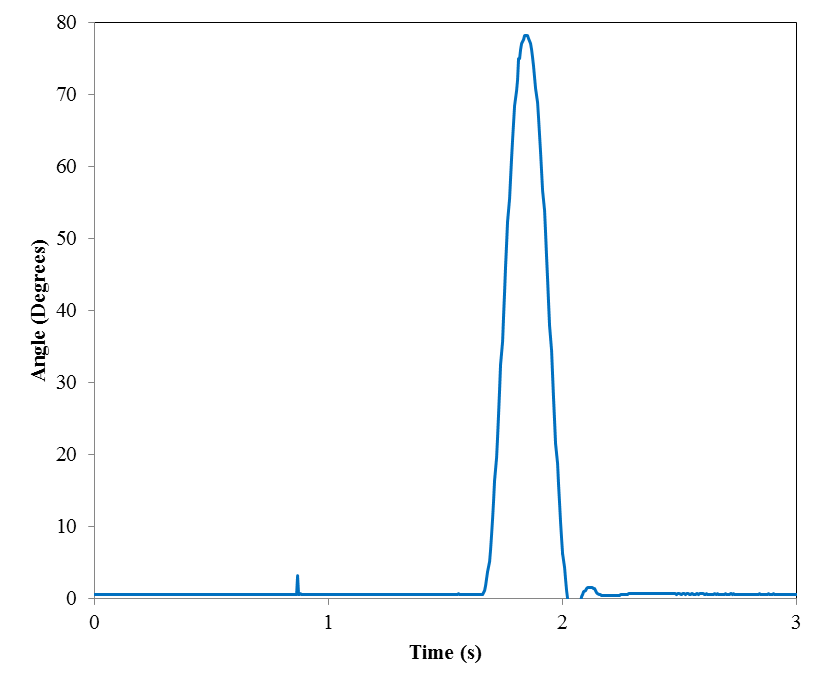

Supplement: Additional file 1: Figure S1 — An angle versus time plot illustrating the footplate traveling through 75° of dorsiflexion. [file 2052-1847-6-14-S1.tiff]
